# A Novel Homozygous Mutation in the KCNJ11 Gene of a Neonate with Congenital Hyperinsulinism and Successful Management with Sirolimus

**DOI:** 10.4274/jcrpe.2773

**Published:** 2016-12-01

**Authors:** Sevim Ünal, Deniz Gönülal, Ahmet Uçaktürk, Betül Siyah Bilgin, Sarah E. Flanagan, Fatih Gürbüz, Meltem Tayfun, Selin Elmaoğulları, Aslıhan Araslı, Fatma Demirel, Sian Ellard, Khalid Hussain

**Affiliations:** 1 Ankara Children’s Hematology-Oncology Training and Research Hospital, Clinic of Neonatology, Ankara, Turkey; 2 Ankara Children’s Hematology-Oncology Training and Research Hospital, Clinic of Pediatric Endocrinology and Metabolism, Ankara, Turkey; 3 University of Exeter Medical School, Biomedical and Clinical Science, Exeter, United Kingdom; 4 Private Doctor; 5 University College London, Department of Pediatric Endocrinology, London, United Kingdom

**Keywords:** congenital hyperinsulinism, newborn, persistent hypoglycemia, sirolimus

## Abstract

Congenital hyperinsulinism (CHI) is the most common cause of neonatal persistent hypoglycemia caused by mutations in nine known genes. Early diagnosis and treatment are important to prevent brain injury. The clinical presentation and response to pharmacological therapy may vary depending on the underlying pathology. Genetic analysis is important in the diagnosis, treatment, patient follow-up, and prediction of recurrence risk within families. Our patient had severe hypoglycemia and seizure following birth. His diagnostic evaluations including genetic testing confirmed CHI. He was treated with a high-glucose infusion, high-dose diazoxide, nifedipine, and glucagon infusion. A novel homozygous mutation (p.F315I) in the KCNJ11 gene, leading to diazoxide-unresponsive CHI, was identified. Both parents were heterozygous for this mutation. Our patient’s clinical course was complicated by severe refractory hypoglycemia; he was successfully managed with sirolimus and surgical intervention was not required. Diazoxide, nifedipine, and glucagon were discontinued gradually following sirolimus therapy. The patient was discharged at 2 months of age on low-dose octreotide and sirolimus. His outpatient clinical follow-up continues with no episodes of hypoglycemia. We present a novel homozygous p.F315I mutation in the KCNJ11 gene leading to diazoxide-unresponsive CHI in a neonate. This case illustrates the challenges associated with the diagnosis and management of CHI, as well as the successful therapy with sirolimus.

**WHAT IS ALREADY KNOWN ON THIS TOPIC?**

Congenital hyperinsulinism is a genetic disorder characterised by challenges associated with the diagnosis and management.

**WHAT THIS STUDY ADDS?**

This case has a novel homozygous p.F315I mutation in the KCNJ11 gene unresponsive to diazoxide therapy but successfully treated with sirolimus before pancreatectomy.

## INTRODUCTION

Congenital hyperinsulinism (CHI) is a complex heterogeneous genetic condition caused by unregulated insulin secretion from pancreatic β-cells. The inappropriate release of insulin leads to persistent severe hypoketotic hypofattyacidemic hypoglycemia and most of the cases present in the neonatal period (1). The prevalence of the disorder is 1 in 40000-50000 live births, increasing to 1:2500 in consanguineous populations (2). Infants are usually macrosomic at birth and require a high glucose infusion rate (GIR) (3). The molecular basis of CHI involves defects in key genes controlling complex mechanisms of insulin secretion. Thus far, mutations in nine genes have been identified and broadly classified into channelopathies and metabolopathies (4). The former are attributed to adenosine triphosphate (KATP)-sensitive potassium channel genes (ABCC8, KCNJ11) and metabolopathies regulate different pathways (GLUD1, GCK, HNF4A, HNF1A, SLC16A1, UCP2, HADH). The most common forms affect KATP channel genes and are predominantly recessive mutations (5,6). There are three histological forms: focal CHI (FCHI) or β-cell adenomatosis, diffuse CHI (DCHI), and atypical CHI (6,7,8,9).

Early recognition, diagnosis, and initiation of immediate management are important in CHI. Maintenance of a normoglycemic state and prevention of permanent brain damage are the principal aims of the treatment (10). Once CHI is confirmed, diazoxide given in a dose of 10-15 mg/kg/d is the drug of choice in medical management (1,5). Fluid retention and hypertrichosis are common side effects of diazoxide, and most centers use chlorothiazide concurrently (1,5). Nifedipine has been used (0.5-2 mg/d), however, the vast majority of patients fail to respond to nifedipine (10). Most of the diazoxide-unresponsive patients have recessive mutations in the ABCC8 or KCNJ11 gene (11). Octreotide may be added to the treatment in doses of 10-50 µg/kg/d. Glucagon in doses of 1-20 µg/kg/h should be used for acute management or in combination with octreotide (10).

The response to diazoxide therapy plays a key role in the management of CHI. Because of inactivating mutations in the ABCC8 and KCNJ11 genes, diazoxide is often ineffective in DCHI and focal forms. The vast majority of patients with DCHI undergo a near-total pancreatectomy, resulting in diabetes and exocrine pancreatic insufficiency (12,13). The indications for surgery include medically unresponsive DCHI and confirmed FCHI by fluoro-18-L-dihydroxyphenylalanine positron emission tomography/computed tomography (18F-DOPA-PET/CT) (5,10,13). Additional therapy with diazoxide, octreotide, and/or frequent feedings may be required postoperatively (14,15).

We present a male infant who was diagnosed with CHI and had a novel homozygous p.F315I mutation in the KCNJ11 gene leading to diazoxide-unresponsive CHI. Both parents were heterozygous for this mutation, and the patient was successfully managed with sirolimus therapy.

## CASE REPORT

This male infant was born to a healthy 26-year-old mother at 37^4/7^ weeks of gestation via cesarean section with Apgar scores 8 and 9 at first and fifth minutes. The infant weighed 4190 g (large for gestational age) at birth. The parents were second cousins and there was no history of a similar condition or of diabetes mellitus in the family. The patient was diagnosed as a case of persistent hypoglycemia and was given GIR up to 14 mg/kg/min and prednisolone 2 mg/kg/d. Serum levels were: insulin 42.5 µU/mL, cortisol 10.9 mg/dL, growth hormone 32.1 ng/mL, and C-peptide 2.7 ng/mL at the time of hypoglycemia (blood glucose level 32 mg/dL). The patient experienced hypoglycemic attacks despite the use of diazoxide (10 mg/kg/d) and octreotide (10 μg/kg/d) and was referred to our unit at age 21 days.

On admission, his physical examination was unremarkable except for macrosomia. The results of his laboratory analysis including a hemogram, acute phase reactants, arterial blood gases, biochemical and urinary evaluations were within normal limits. Echocardiography revealed an atrial septal defect and mild septal hypertrophy. The patient was administered enteral nutrition with breast milk at 2-h intervals. Despite intensive therapy (diazoxide 25 mg/kg/d, chlorothiazide 1 mg/kg/d, octreotide 40 μg/kg/d, glucagon infusion 10 μg/kg/h, and nifedipine 1 mg/kg/d), his hypoglycemia persisted until 1 month of age. As ^18^F-DOPA-PET/CT was unavailable, we could not determine the histological form of the disorder. DNA samples were sent to the United Kingdom (Exeter Clinical Laboratory, Exeter, UK) for mutation analysis.

We considered sirolimus therapy before pancreatectomy when the patient was 35 days old. Parental consent and approval of off-label use of the drug from the Turkish Ministry of Health Ethics Committee were obtained. His glucose levels increased following oral sirolimus therapy in a dose of 0.5 mg/m2/d. We stopped diazoxide, hydrochlorothiazide, nifedipine, and glucagon infusion gradually, and his octreotide dose was decreased to 5 μg/kg/d. The sirolimus dose was adjusted according to the serum level. He was discharged at 2 months of age with pre-feed home blood glucose monitoring on low-dose octreotide (5 μg/kg/d) twice-daily subcutaneous injections and sirolimus (0.3 mg/m^2^/d). His outpatient clinical follow-up continued without hypoglycemia. At the time of this report, the patient was 5 months of age, there were no abnormal findings on his neurological or physical examination and cranial magnetic resonance imaging revealed normal findings according to his age.

Genetic analysis revealed a novel homozygous mutation in the KCNJ11 gene (p.F315Ic.943T>A); the parents were heterozygous for this mutation. This mutation had not been previously identified in over 3000 patients with CHI or with neonatal diabetes referred to the Exeter Clinical Laboratory for testing.

## DISCUSSION

CHI is a heterogeneous disorder caused by mutations in nine key genes regulating insulin secretion. The ABCC8 and KCNJ11 genes (both localized on chromosome 11p15.1) encode the two components of the K_ATP_ channel: the pore-forming inward rectifier potassium channel subunit (KIR6.2) and the regulatory subunit sulfonylurea receptor 1 (SUR1) (1,2,3,4,5,6). Loss-of-function mutations in these genes are present in the homozygous or compound heterozygous state and may be dominantly acting. Recessive inactivating mutations in the ABCC8 and KCNJ11 genes constitute the most common and severe forms of CHI. Approximately 300 different mutations in ABCC8 and 30 mutations in KCNJ11 account for 36.3% of all cases (12,16,17). Genetic analysis in our patient revealed a novel homozygous mutation in the KCNJ11 gene (p.F315I) leading to diazoxide-unresponsive DCHI.

Although the molecular mechanisms of FCHI and DCHI are different, their clinical presentations appear to be similar. DCHI is inherited in an autosomal recessive manner in most cases, whereas FCHI is sporadic (12,16). It is well known that patients with homozygous recessive or compound heterozygous mutations in the ABCC8 or KCNJ11 gene present with DCHI. They are usually medically unresponsive and account for 60-70% of all cases. Therefore, our diazoxide-unresponsive patient may have DCHI. Paternally inherited mutations in the ABCC8 or KCNJ11 gene and a concomitant loss of the maternal 11p allele (11p15.1-11p15.5) result in focal pancreatic lesions (6,9,10,12,16).

The management of diazoxide-unresponsive DCHI constitutes a major therapeutic challenge. Because of abnormal activation of the mammalian target of rapamycin (mTOR) pathway in several neoplasms, including insulinoma, mTOR inhibitors have been increasingly recognized as a treatment option in patients with CHI (18,19). Senniappan et al (20) described four infants with severe CHI unresponsive to maximal doses of diazoxide (20 mg/kg/d) and octreotide (35 μg/kg/d). They reported that all patients showed a clear glycemic response to sirolimus, but one patient required a small dose of octreotide to maintain normoglycemia. Abraham described a neonate with CHI caused by a homozygous ABCC8 mutation, who was unresponsive to diazoxide and octreotide (21). He reported achievement of euglycemia by using sirolimus therapy postoperatively. Kara et al (22) treated a newborn having CHI with sirolimus, but discontinued it due to hepatic and renal failure. Another report describes an 8-year-old boy with severe CHI due to a biallelic heterozygous ABCC8 mutation who exhibited a drastic improvement with sirolimus (23). Sirolimus therapy appears to be a feasible alternative to subtotal pancreatectomy, either alone or in combination with somatostatin analogues for selected patients with no contraindications. Sirolimus allows the discontinuation of intravenous dextrose, glucagon infusion, and octreotide. In our patient, following sirolimus therapy, ‘we discontinued diazoxide, thiazide, glucagon, and glucose infusion and decreased the dose of octreotide.

The adverse effects of mTOR inhibitors, such as everolimus and sirolimus, include stomatitis, increased risk of infection, immunosuppression, abnormalities in renal function, fatigue, and pneumonitis. Transient elevations of aminotransferase levels have been reported. Patients with CHI who receive sirolimus therapy must be monitored regularly to assess glycemic control and adverse events (20,24). During the short follow-up period, we did not observe any adverse effects caused by sirolimus.

Patients with CHI should be closely followed to monitor the efficacy of treatment and complications related to medications and underlying disease. These patients should record their blood glucose levels using a home glucometer. Blood glucose level, diet, growth, and side effects of medications should be regularly evaluated (2,25,26). It is clear that early severe hypoglycemic events may cause poor neurological outcomes, such as psychomotor retardation, cognitive deficit, epilepsy, and cerebral palsy. Neonatal onset of CHI is usually more severe and requires regular screening, detection, and appropriate management by a pediatric neurologist (27,28,29). We trained the mother of our patient in home glucose monitoring and arranged visits with a pediatric endocrinologist and neurologist following discharge. Our patient had normal neurodevelopment at 5 months of age.

In conclusion, we described the case of a neonate with a novel homozygous KCNJ11 mutation leading to diazoxide-unresponsive DCHI. This case illustrates the pitfalls and challenges associated with the treatment of CHI, as well as the successful therapy with sirolimus.

## Ethics

Informed Consent: It was taken.

Peer-review: Externally peer-reviewed.
